# Advances in WO_3_-Based Supercapacitors: State-of-the-Art Research and Future Perspectives

**DOI:** 10.3390/nano13081418

**Published:** 2023-04-20

**Authors:** Giacometta Mineo, Elena Bruno, Salvo Mirabella

**Affiliations:** 1 Dipartimento di Fisica e Astronomia “Ettore Majorana”, Università degli Studi di Catania, via S. Sofia 64, 95123 Catania, Italy; giacometta.mineo@dfa.unict.it (G.M.); elena.bruno@dfa.unict.it (E.B.); 2 CNR-IMM, Università di Catania, via S. Sofia 64, 95123 Catania, Italy

**Keywords:** energy storage, electrochemical characterization, WO_3_, pseudocapacitor, asymmetric supercapacitor, symmetric supercapacitor

## Abstract

Electrochemical energy storage devices are one of the main protagonists in the ongoing technological advances in the energy field, whereby the development of efficient, sustainable, and durable storage systems aroused a great interest in the scientific community. Batteries, electrical double layer capacitors (EDLC), and pseudocapacitors are characterized in depth in the literature as the most powerful energy storage devices for practical applications. Pseudocapacitors bridge the gap between batteries and EDLCs, thus supplying both high energy and power densities, and transition metal oxide (TMO)-based nanostructures are used for their realization. Among them, WO_3_ nanostructures inspired the scientific community, thanks to WO_3_’s excellent electrochemical stability, low cost, and abundance in nature. This review analyzes the morphological and electrochemical properties of WO_3_ nanostructures and their most used synthesis techniques. Moreover, a brief description of the electrochemical characterization methods of electrodes for energy storage, such as Cyclic Voltammetry (CV), Galvanostatic Charge–Discharge (GCD), and Electrochemical Impedance Spectroscopy (EIS) are reported, to better understand the recent advances in WO_3_-based nanostructures, such as pore WO_3_ nanostructures, WO_3_/carbon nanocomposites, and metal-doped WO_3_ nanostructure-based electrodes for pseudocapacitor applications. This analysis is reported in terms of specific capacitance calculated as a function of current density and scan rate. Then we move to the recent progress made for the design and fabrication of WO_3_-based symmetric and asymmetric supercapacitors (SSCs and ASCs), thus studying a comparative Ragone plot of the state-of-the-art research.

## 1. Introduction

**Figure 1:**
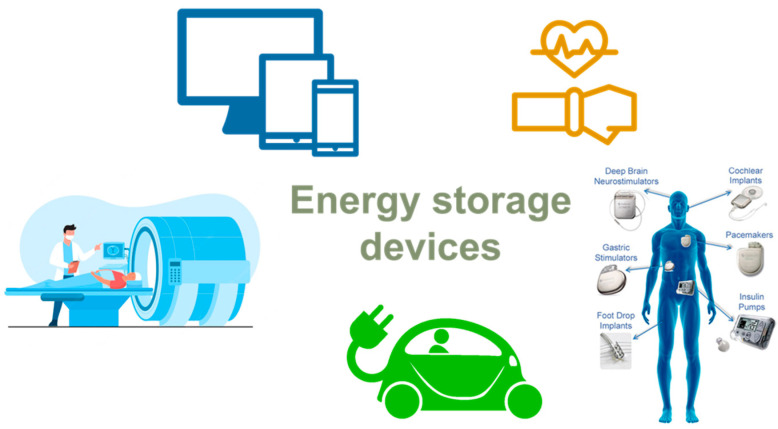
Examples of the emerging applications for which the development of efficient energy storage devices are crucial.

In the global panorama, a great interest in the scientific community is addressed to green and renewable sources of energy, to mitigate issues such as CO_2_ emissions and the finite supply of fossil fuels. In this scenario, the development of efficient energy storage devices is decisive to make actual use of renewable energy sources, such as wind, hydropower, Sun, and heat [1,2]. Their role is to collect the excess energy during production, act as reservoirs or carriers, and release the energy when and where needed [3]. Energy storage devices have been around for a long time and were used for many different applications, even if related environmental concerns were neglected. The technological development we are experiencing has made modern electronic devices portable and compact in a user-friendly way and requires high performance energy storage devices in terms of durability, efficiency, and sustainability (Figure 1).

**Figure 2:**
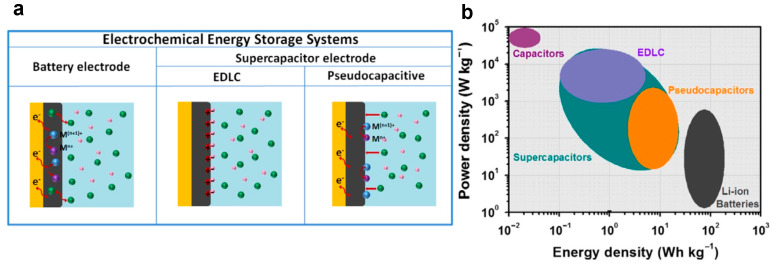
(**a**) Schematic of the energy storage mechanism of batteries and supercapacitors (EDLC and pseudocapacitors); (**b**) Ragone plot (specific power density against specific energy density) of energy storage devices. Reproduced with permission by [4].

In the panorama of electrochemical energy storage systems, batteries, and supercapacitors (Electrical Double Layer Capacitors, EDLCs, and pseudocapacitors) are used depending on the power and energy density required by applications. These devices mainly differ in the energy storage mechanisms occurring at the surface of the electrochemically active material. Figure 2a shows a schematic representation of the energy storage mechanisms at the microscopic scale, which are related to the chemical reactions at the electrode surface for batteries and supercapacitors [4]. In batteries, the charge (discharge) mechanism occurs by the intercalation (de-intercalation) of the electrolyte cations (H^+^ or Li^+^) in the bulk of the material where they react with redox reactions (diffusion-limited charge storage mechanisms). Batteries are the better choice when high energy density is required since the energy storage mechanism involves almost the whole active material. Nevertheless, the intercalation and the deintercalation of the cations occur slowly, so the power density supplied by a battery can be limited [5]. In Electrical Double Layer Capacitors, the charge and discharge mechanisms occur through the adsorption and the desorption of cations or anions in correspondence with surface-active sites at the electrode–electrolyte interface (surface-limited charge storage mechanisms). Indeed, EDLCs represent a promising solution in high-power density applications, since the electrochemical reactions driving the energy storage mechanisms occur at the surface of the active material, avoiding the drawbacks related to ion intercalation. Notwithstanding, only a small percentage of the total active material is available for electrochemical reactions, so the energy density supplied by an EDLC is typically lower than in batteries [6].

To overcome the drawbacks of EDLC and batteries, a new device, called a pseudocapacitor, has been recently developed. In pseudocapacitors, the energy storage mechanism occurs through the adsorption and the desorption of cations and anions at the surface of the electrochemically active material, coupled with shallow redox reactions. This results in high levels of quickly available stored charge, which allows supplying high power and energy density at the same time with a single device [7,8]. Indeed, a pseudocapacitor works as a battery, but redox reactions occur at the surface, such as in an EDLC [4], thus joining the intermediate power and energy density of both kinds of devices. This behavior results in a combination of diffusion and surface-limited charge storage mechanisms, which can be individuated by cyclic voltammetry (CV) analysis [9]. Figure 2b shows the Ragone plot where electrochemical energy storage devices are reported as a function of energy and power density. Following the above, EDLCs supply high power density and low energy density (violet region), batteries supply low power density and high energy density (grey region), and pseudocapacitors are in the middle (orange region) [4].

Pseudocapacitors aroused a great interest in the scientific community since they are suitable candidates for the development of high-capacitance energy storage devices. They are typically obtained by using Li-Na metal compounds [10], metal-based biomass-derived carbon composites [11], and highly porous transition metal oxide-based electrodes, based on MnO [12], CoO [13], RuO_2_ [14] ZnO [15], SnO_2_ [16], CuO [17], TiO_2_ [18], and WO_3_ [19,20,21]. Among them, WO_3_ has emerged as a favorable material thanks to its properties, especially in the nanostructured form. The energy storage mechanism mainly occurs at the electrode–electrolyte interface and strongly depends on the exposed electrode surface, which can be properly modified with the help of nanotechnology. The introduction of nanotechnology leads to an improvement in electrochemical activity since nanoscale materials show unique physical and chemical properties compared with their bulk state, depending on their unique shape and size [22]. In fact, for the nanostructured form, it is possible to obtain [23]:

- A greater surface-to-volume ratio compared to bulk form, providing more surface area for physical and chemical interactions;
- Quantum confinement effects due to the small size of nanostructured forms that influences optical properties, electronic band structure, and electrical charge transport;
- Significantly altered surface energy, which can be used to modify the bond structures of atomic species close to the surfaces.

Nanoparticles, nanorods, and nanobundles can be considered as 0, 1, and 2-D nanostructures, respectively, and possess different physical and chemical properties because of changes in surface-to-volume ratio and charge confinement [24,25]. One of the most important advantages of nanotechnology is the chance to properly tailor the crystalline phase and morphology of nanostructures, to affect physical and chemical properties, to improve electrochemical activity. Moreover, the high surface-to-volume ratio of nanostructures allows a reduction in the mass of the used active material. This aspect is crucial within a life cycle assessment analysis, taking into consideration recycling and decreasing waste, or in cases in which noble metals or critical raw materials (CRMs) are used [26]. The use of nanostructures contributed to the brilliant recent scientific results on WO_3_ for energy storage, electrochromism, photocatalytic, and sensing applications [19,27,28,29,30,31,32,33,34].

Herein, a review of the state-of-the-art research about electrochemical energy storage activity of nanostructured WO_3_ is reported. The properties of WO_3_ are analyzed from the perspective of energy storage improvement, focusing on the specialized synthesis of performing nanostructures to realize competitive pseudocapacitors. After a general introduction for beginners on electrochemical analysis of energy storage performances, several approaches used to prepare electrodes are thoroughly summarized and commented, and recent progresses in the electrochemical performances of WO_3_ and WO_3_-based composites are reported. Indeed, a short assessment of the realization of WO_3_-based asymmetric and symmetric supercapacitors is presented.

## 2. WO_3_: Key Material for Energy Storage

### 2.1. Crystal Structure Properties

WO_3_ is an *n*-type semiconductor with high electrochemical stability in acidic environments, and high intrinsic density (>7 g∙cm^−3^) [35]. Its energy storage performances strongly depend on the crystal structure, which can make the ions’ intercalation easier in an electrochemical environment.

**Figure 3:**
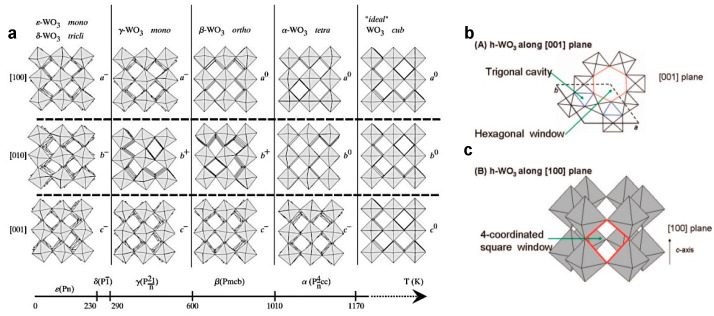
(**a**) Tilt patterns and stability temperature domains of the different polymorphs of WO_3_; (**b**) the structure of h-WO_3_ shown with the *c*-axis perpendicular and (**c**) parallel to the plane. Reproduced by [24].

In its crystalline form, WO_3_ is made of octahedra sharing corners and edges, where each W atom is linked to six O atoms, as Figure 3 shows [24]. Thanks to the high coordination number, WO_3_ possesses many crystalline phases, which depend on the rotation direction and tilting angles of the WO_6_ octahedra (Figure 3a) with respect to the ideal cubic perovskite-like structure, whose stability depends on temperature [30]. The hexagonal phase is metastable, and it is turned into a monoclinic I phase when the temperature is higher than 400 °C [24,27]. A unique feature of h-WO_3_ is that WO_6_ octahedra share corner oxygen atoms in three- and six-membered ring arrangements along the (001) plane. This sharing forms three different types of tunnels in the W-O bulk structure, which are triangular and hexagonal cavities along the *ab* plane and square windows along the *c* axis, as shown in Figure 3b,c. According to the literature, these cavities can act as preferential ions intercalation channel for applications in electrochemical environment [30].

### 2.2. WO_3_ Nanostructure Synthesis Approaches

The nanotechnology advantages in a multitude of applications made the large-scale synthesis of nanostructures a crucial point for the development of new promising technologies. The electrochemical activity of WO_3_ nanostructures towards energy storage strongly depends on the morphology and crystal structure, and consequently, on the synthesis techniques. WO_3_ can be easily synthesized in a nanostructured form by different approaches, such as Vapor-Phase and Liquid-Phase Synthesis.

**Figure 4:**
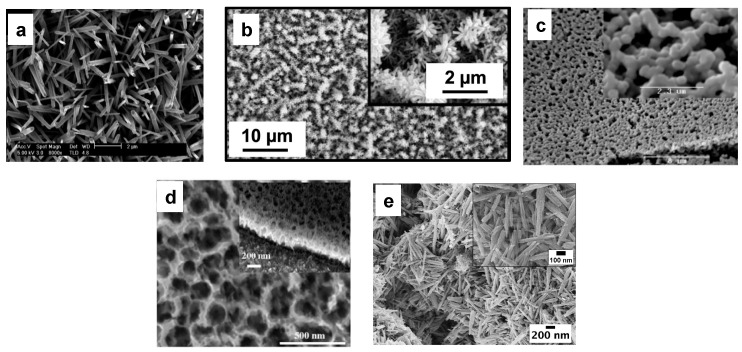
SEM images of WO_3_ nanostructures (nanowires, nanorods, mesoporous and nanostructured film, nanospheres, and nanorods, respectively) synthesized (**a**) by thermal evaporation [36]; (**b**) by hot wire CVD [37]; (**c**) by sol-gel method [38]; (**d**) by electrochemical anodization [39]; and (**e**) by hydrothermal synthesis [29]. Reproduced with permission.

The Vapor-Phase Synthesis involves the condensation of a vaporized source material onto the substrate, using an expensive experimental setup [24]. Two types of deposition can be distinguished: Physical Vapor Deposition (or PVD) and Chemical Vapor Deposition (or CVD). Baek et al. [36], synthesized a dense WO_3_ nanowire film on a W substrate by thermal evaporation (Figure 4a). Shankar et al. [37] synthesized WO_3_ nanorods by using a hot filament chemical vapor deposition (HFCVD) with carbon nanotubes as a template (Figure 4b). For practical application, the low-cost, large-scale synthesis of nanostructures is necessary. In this scenario, Liquid Phase Syntheses, such as sol-gel, electrochemical anodization, and hydrothermal, are very attractive being characterized by simple equipment, low costs, and high reproducibility. Room temperatures are compatible with these processes, and good control and reproducibility can be achieved. Peroxotungstic acid (H_2_W_2_O_11_) is generally used as a precursor for the WO_3_ synthesis, thanks to its high stability at room temperature and in an acidic environment [24]. Yang et al. [38] synthesized mesoporous WO_3_ film by using a simple sol-gel route (Figure 4c). Electrochemical anodization is widely used for the industrial synthesis of metal oxide films, thanks to its simplicity. Zheng et al. [39] synthesized a nanostructured WO_3_ film by using a typical anodization route with a W foil as the anode (Figure 4d). Unfortunately, the high voltages required for the synthesis and the difficulty to achieve the desired nanostructured morphology make the anodization technique difficult to perform for the WO_3_ nanostructure synthesis. The hydrothermal procedure represents one of the greenest, simplest, and most versatile procedures among all the Liquid Phase Synthesis methods viable for the synthesis of WO_3_ nanostructures. It does not require any external potential and the preparation of the precursor solution occurs in just a few steps. Nanostructure formation can occur both in high and low temperature and pressure conditions. Moreover, the morphology and crystallinity of nanostructures strongly depend on precursor solution components, and on reaction time and temperature [40]. For example, Mineo et al. [29] synthesized WO_3_ nanorods by using a simple hydrothermal route with NaCl as the capping agent (Figure 4e), which confines the growth along the *c*-axis.

### 2.3. Affinity of WO_3_ for Energy Storage Applications

WO_3_ nanostructures possess structural flexibility, stability in an acidic environment, and resistance to electrochemical corrosion, which makes it a suitable candidate for electrochemical energy storage. The electrochemical reactions occur at the electrode surface and involve electron and ion transfer, so high exposed surface and good conductivity are preferable and the optimization of several factors, such as specific surface area and mass loading, affect the energy storage activity of WO_3_. Unfortunately, stoichiometric WO_3_ is characterized by poor electron conductivity, which can be improved by properly tailoring the morphology and crystallinity of WO_3_-based nanostructures, or by using carbon-based nanocomposites during the electrode preparation [41,42,43,44,45]. It was demonstrated that in comparison to other polymorphisms, 1D hexagonal WO_3_ nanostructures possess the highest energy storage performances, thanks to the presence of triangular and hexagonal cavities and square windows in the crystal structure (Figure 3b). These tunnels can provide accommodation sites for many cations during the electrochemical process, by facilitating electrolyte ion insertion and storage in the WO_3_ matrix thanks to its multiple oxidation states [30].

State-of-the-art research on WO_3_ demonstrates that it exhibits a pseudocapacitor behavior with quasi-rectangular cyclic voltammetry (CV) curves [19,42,44,46,47]. According to Dunn et al. [9] the charge storage mechanism in WO_3_ can be described in terms of surface and diffusion-limited contributions and occurs at the electrode–electrolyte interface. Surface-limited contributions are related to the adsorption/desorption of charge on the surface, while diffusion-limited contributions result from redox reactions that occur at the surface during which the W oxidation state changes as follows [48]:

$${\mathrm{WO}}_{3}+{\mathrm{xM}}^{+}+{\mathrm{xe}}^{-}⇌M_{x}{\mathrm{WO}}_{3-x}$$

in which M^+^ represents the cation of the used electrolyte (H^+^, Na^+^, Li^+^).

WO_3_ is characterized by many oxidation states, which promote redox reactions at the active material surface. The high theoretical capacitance and the possibility to easily tailor the morphological and crystal properties of WO_3_ make it a suitable candidate for the development of an efficient anode in energy storage devices, such as symmetric and asymmetric supercapacitors. These WO_3_ features have aroused a great interest in the scientific community, and many efforts have been made to pave the way to the development of very efficient WO_3_-based energy storage devices.

## 3. WO_3_-Based Electrode Preparation

**Figure 5:**
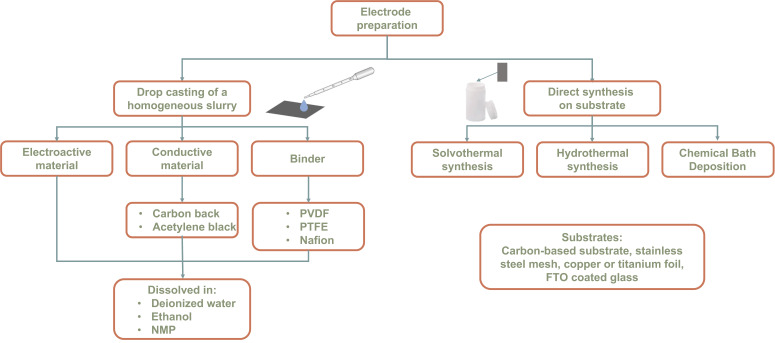
Schematic description of the different methods for electrode preparation.

A solid comparison among different electrodes for energy storage cannot neglect the preparation methods since electrode realization details can impact the final performance of WO_3_. The energy storage mechanism is activated by an electric field, thus the contact between the active material and a proper substrate is a key point in preparing an electrode. Below, a review of the most used procedure for WO_3_ electrode preparation is reported. The most used substrates are stainless steel mesh [43,49], copper [50,51] or titanium foil [52], fluorine tin oxide (FTO) coated glass [53,54], and carbon-based substrates [44,45]. State-of-the-art research reports two different methods for the electrode realization: the deposition (e.g., by drop casting or spin coating) of a homogeneous WO_3_-based slurry, or the direct synthesis of the active materials onto the electrode surface, as Figure 5 shows.

In most cases, the homogeneous slurry is prepared by mixing different concentrations of electroactive material (WO_3_ nanostructures for instance) with a conductive material (carbon black or acetylene black) and a binder (Nafion, polyvinylidene fluoride PVDF, or polytetrafluoroethylene PTFE). The conductive material is used to improve the electron conductivity of the electrochemically active material [44], while the binder acts as a dispersion agent, to improve the adhesion with the substrate and to link the nanostructures together [55]. Moreover, to improve the homogeneity of the slurry, the electroactive material-conductive material-binder dispersion is mixed with different solvents, such as deionized water, ethanol, and N-Methyl-2-pyrrolidone (NMP). Lockande et al. [42] prepared the electrode by drop casting on carbon cloth a homogeneous WO_3_-based slurry, prepared by dissolving 3 mL of Nafion into a mixture of WO_3_ nanorods, carbon black, and PVDF with a 80:5:15% concentration, respectively; Shi et al. [43] prepared the electrode by drop casting on a stainless steel grid an ethanol dispersion which contains hierarchical porous lignin-derived carbon (HPC)/WO_3_ nanostructures, acetylene black, and PTFE with an 8:1:1% concentration; Nayak et al. [56] painted a graphite sheet electrode with an homogeneous slurry obtained by mixing WO_3_/graphene nanocomposites and PVDF in 2 mL of NMP; Jia et al. [44] used a glassy carbon substrate coated with a homogeneous slurry prepared by mixing WO_3_ nanostructures, carbon black, and PVDF (80:10:10%) in a certain amount of NMP; Liu et al. [45] prepared an homogeneous slurry by mixing WO_3_, carbon black, and PVDF (8:1:1%) in NMP, which was dropped on a carbon cloth substrate; Shao et al. [52] drop cast onto a titanium current collector a homogeneous slurry prepared by mixing WO_3_, acetylene black, and PTFE (70:20:10%) in ethanol. Despite the effort of the scientific community for the determination of the well-optimized homogeneous slurry composition, the main problems of electrodes to be prepared are related to the adhesion of nanostructures on the substrate and their stability. To overcome this problem, electrodes are prepared by direct synthesis of active materials on the substrate. The adhesion of the active material strongly depends on the synthesis technique. The most used are the solvothermal and the hydrothermal synthesis, the chemical bath deposition, and the electrodeposition, as Figure 5 shows. The substrate is immersed in the precursor solution during the synthesis, and the nanostructure growth occurs on the surface, thus drastically reducing the problems related to electrical resistance due to the interface between the electroactive material to the substrate. The hydrothermal route is the most used technique for the synthesis of nanostructures directly on the electrode surface: Shinde et al. [57] synthesized WO_3_ nanorods on carbon cloth; Zheng et al. [50] synthesized a hexagonal WO_3_ nanoflake array directly on a copper foil substrate; Ji et al. [46] synthesized WO_3_ nanorods directly on a carbon cloth substrate; He at al. [49] synthesized three-dimensional hierarchical furball-like WO_3_ nanospheres on a stainless steel mesh; Gao et al. [58] synthesized WO_3_ nanowires on a carbon cloth substrate; Huang et al. [59] synthesized 1D, 2D, and 3D WO_3_ nanostructures on a stainless steel substrate. Additionally, solvothermal synthesis is used for the direct synthesis of the electroactive material on the electrode surface: Jung et al. [60] grew W_18_O_49_ and WO_3_ nanowires directly on carbon felt substrate; Su et al. [53] synthesized WO_3_ nanowires on FTO-coated glass with a seed layer. Only a few articles report the synthesis of WO_3_ nanostructures by using the chemical bath deposition as described by Shinde et al. [61], which synthesized monoclinic WO_3_ directly on a stainless-steel substrate.

## 4. WO_3_ for Energy Storage

WO_3_ nanostructures, such as nanorods and nanowires, were widely studied in the literature for energy storage application. In the following, a brief review on the state-of-the-art research about recent progress obtained by using WO_3_-based electrodes is reported. Data will be properly grouped into three main categories, which reflect the main strategies to improve the energy storage ability of WO_3_. First, state-of-the-art research on energy storage by bare WO_3_ nanostructures will be presented, followed by recent progress obtained by using WO_3_–carbon-based nanocomposites and metal-doped WO_3_ nanostructures. The energy storage performances of different WO_3_-based electrodes will be compared in terms of the specific capacitance calculated from CV and GCD curves, respectively, as follows [5]:

(1) $$C_{s}=\frac{\int IdV}{m\upsilon \Delta V}$$

(2) $$C_{s}=\frac{It_{s}}{m\Delta V}$$

where *I* is the measured current (mA), *V* is the measured potential (V), *m* is the total mass of the active material (mg), *υ* is the voltage scan rate (V/s) and ∆*V* is the voltage scanned window (V), and *t_s_* is the discharge time.

### 4.1. WO_3_ Nanostructures for Pseudocapacitors

WO_3_ nanostructures represent outstanding and low-cost candidates for energy storage applications and aroused a large interest in the scientific community. Table 1 shows the most important parameters of some of the WO_3_-based electrodes reported in the literature for energy storage performances. Their energy storage activity was mainly studied in acidic conditions (sulfuric acid, H_2_SO_4_, is the most used electrolyte) as a function of different parameters, such as morphology, crystal structure, stoichiometry to optimize the performance in terms of C_s_, calculated from CV and GCD analysis. The role of the WO_3_ nanostructure morphology and crystal structure in energy storage activity was studied by Lockande et al. [42], who synthesized different WO_3_ nanostructures (nanocubes, nanorods, and nanoplates) with different crystal structures (hexagonal, monoclinic, orthorhombic, and tetragonal) using the hydrothermal method and by varying the pH of the precursor solution and the synthesis temperature. The authors described the energy storage mechanism in terms of surface capacitive and diffusion-limited contributions, thus confirming that the hexagonal and the orthorhombic crystal structures are the most suitable for pseudocapacitor applications. Moreover, they found that the hexagonal WO_3_ nanocubes showed the highest C_s_ of 377 F/g at 2 mV/s, unlike the monoclinic nanocubes, which showed the lowest C_s_ of 325 F/g at 2 mV/s. The crystal quality role in energy storage performances was also studied by Zheng et al. [50]. The authors proposed a simple hydrothermal route to synthesize single-crystal, polycrystal, and hierarchical hexagonal WO_3_ nanoflakes, thus demonstrating the pseudocapacitor behavior of WO_3_ nanoflakes, regardless of the crystal composition. CV and GCD analysis confirm the superiority of the hierarchical hexagonal WO_3_ nanoflakes, which possess the highest C_s_ (588 F/g at 5 mV/s and 538 F/g at 0.1 A/g) and the smallest charge resistance. The authors ascribed the superior performances of the hierarchical structure to the distribution of oxygen atoms in the crystal lattice, whose position is favorable for oxygen vacancy creation, which improves electronic transport.

The introduction of phase junctions in WO_3_ nanostructures can be a suitable strategy to improve energy storage performance. Liu et al. [45] synthesized WO_3_ nanotubes on nanoplates with a hexagonal/orthorhombic heterophase structure by using the hydrothermal technique. The surface and diffusion contributions were evaluated starting from the analysis of the CV curves at different scan rates. The hexagonal/orthorhombic WO_3_ nanoplates possess high areal capacitance (2552 mF/cm^2^ at 1 mA/cm^2^), compared with that of commercial WO_3_ nanoplates (742 mF/cm^2^ at 1 mA/cm^2^).

Beyond morphology and crystal structure, the energy storage performances also depend on stoichiometry. Jung et al. [60] used hydrothermal synthesis to synthesize W_18_O_49_ nanowires on carbon felt substrate which becomes WO_3_ nanowires after thermal treatment at 500 °C for 10 h. The energy storage performances of the stoichiometric and of the non-stoichiometric phase were compared, demonstrating that the presence of oxygen vacancy in W_18_O_49_ increases the number of W^5+^ and W^4+^ states, facilitating the ion insertion process. As a result, a higher C_s_ is obtained for W_18_O_49_ nanowire-based electrodes compared to that obtained for WO_3_ nanowires (550.8 and 448.8 F/g at 10 mV/s, respectively). The optimization of morphological and structural properties of WO_3_ nanostructures is necessary but not crucial to guarantee high performance since the latter also depends on the polishing procedure of WO_3_-based electrodes. Jia et al. [44] studied the role of the cleaning procedure to remove the residual ions of the precursor solution in the energy storage performance of WO_3_ nanoplates synthesized by the hydrothermal method. They demonstrated that after the cleaning procedure, the C_s_ of WO_3_ nanoplates increases from 203 F/g to 334 F/g at 2 mV/s.

**Figure 6:**
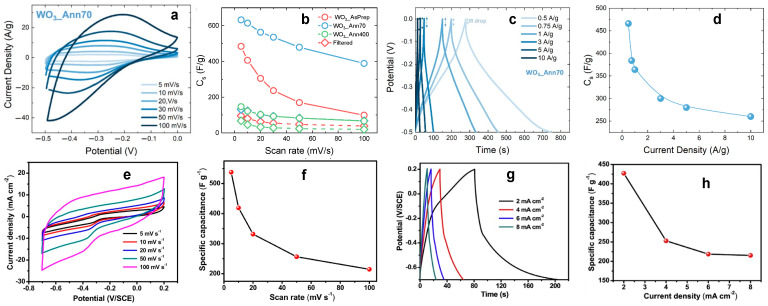
(**a**,**e**) CV and (**c**,**g**) GCD curves and the related Cs as a function of (**b**,**f**) scan rate and (**d**,**h**) current density, respectively, of (**a**–**d**) hexagonal WO_3_ nanorods and urchin-like nanostructures [19] and (**e**–**h**) hexagonal WO_3_ nanorods [57]. Reproduced with permission.

Chemical reactions which occur at the electrode surface leading to energy storage strongly depend on the morphology and crystal structure of WO_3_-based electrodes, whereby the CV and GCD curves can be drastically different despite the similarity of the measurement conditions. Figure 6 shows the comparison between CV and GCD curves and related C_s_ as a function of scan rate and current density curves of two different WO_3_-based electrodes, tested with the same electrolyte (1 M H_2_SO_4_). Figure 6a–d was obtained after the electrochemical analysis of hexagonal WO_3_ nanorods and urchin-like nanostructures on a graphene paper substrate [19], while Figure 6e,f is related to the electrochemical analysis of hexagonal WO_3_ nanorods on a carbon cloth [57]. In both cases, hydrothermal synthesis is used for the synthesis of WO_3_ nanostructures. Mineo et al. [19] prepared a WO_3_-based homogeneous slurry and drop-coated some drops on the conductive substrate for the electrode preparation, while Shinde et al. [57] synthesized WO_3_ nanorods directly on the carbon cloth. Both electrodes show a pseudocapacitor behavior, as the shape of CV (Figure 6a,c) and GCD (Figure 6e,g) curves reveal. The trend of the specific capacitance as a function of scan rate (Figure 6b,d) and of current density (Figure 6f,g) confirms that the energy storage mechanism and the pseudocapacitive behavior is the same for both electrodes, regardless of the electrode preparation methods. Moreover, comparable values of C_s_ are obtained from CV (632 F/g and 538 F/g at 5 mV/s) and GCD analysis (466 F/g at 0.5 A/g and 425 F/g at 2 mA/cm^2^) for the electrodes prepared with the homogeneous slurry [19] and the electrodes directly synthesized on a substrate [57]. These results highlight the stable and optimal energy storage performance of WO_3_ nanostructures.

Well-optimized WO_3_ nanostructures in terms of morphology, crystal structure, and stoichiometry allow the achievement of very high energy storage performances, as reported by several recent papers. Wu et al. [62] synthesized WO_3_ nanotube bundles directly on a carbon cloth substrate by hydrothermal procedure, thus obtaining C_s_ of 600 F/g at 3 mA/cm^2^ and cyclic durability of 85% after 6000 cycles of charge–discharge. The outstanding pseudocapacitive activity was obtained by Ji et al. [46], which synthesized hexagonal WO_3_ nanorods directly on a carbon cloth substrate by using hydrothermal synthesis, thus obtaining very high C_s_ of the order of 900 F/g at 5 mV/s. Comparable performances were also obtained by testing WO_3_-based electrodes prepared by using a homogeneous slurry, as Xu et al. demonstrated [63]. The authors synthesized mesoscopic WO_3_ microspheres composed of self-assembly hexagonal nanofibers by hydrothermal route, by obtaining C_s_ of 872.73 F/g at 10 mV/s and 797.05 F/g at 0.5 A/g.

State-of-the-art research on energy storage performances of WO_3_ nanostructures demonstrates the huge potentiality of this material. Low-cost and simple synthesis techniques can be used to tailor the morphology, the crystal structure, and the stoichiometry of nanostructures, thus achieving exceptional performances, without the use of toxic and dangerous precursor materials. Despite the reasonable performances of WO_3_ nanostructures when used for energy storage applications, the problems related to electronic conductivity are challenging to solve. To this aim, different approaches were reported in the literature, such as the preparation of WO_3_–carbon-based nanocomposites, whose recent advances will be reported in the next paragraph.

### 4.2. WO_3_–Carbon-Based Nanocomposites for Pseudocapacitors

WO_3_ nanostructures are a suitable anode candidate for energy storage performances, which strongly depend on morphology, crystal structure, and stoichiometry. The hydrothermal route is the most used since it allows the tailoring of nanostructures in a simple, low-cost, and efficient way. As mentioned above, despite the attempt of the scientific community, the electronic transport properties of WO_3_ remain the weak point for obtaining very efficient devices. The development of hybrid WO_3_–carbon-based nanocomposites is a suitable strategy to improve the electronic conductivity in WO_3_. WO_3_–carbon-based nanocomposites are commonly synthesized by using a solvothermal method since non-aqueous solutions are used for their stable and homogeneous formation. One of the advantages of the use of WO_3_–carbon-based nanocomposites is to exploit the porous structure with which the carbon composites are equipped. Shi et al. [43] solvothermal synthesized, by using a solvothermal method, a three-dimensional hierarchical porous lignin-derived carbon (HPC)/WO_3_ hybrid structure with different carbon compositions. The porous structure and the strong contact between HPC and WO_3_ increase the active sites for the electrochemical surface process and provide a short ion diffusion path. As a consequence, the hybrid structure with the highest carbon content possesses exceptional C_s_ value compared with that of the bare WO_3_ electrode (432 F/g and 214 F/g at 0.5 A/g, respectively).

Among the carbon-based materials, graphene is a zero-gap semiconductor [64] able to confer high electrochemical stability and exceptional electronic conductivity when coupled with WO_3_ nanostructures. The interaction between WO_3_ nanostructures and graphene was investigated in-depth in the literature, by confirming the outstanding properties of these nanocomposites. Guan et al. [65] synthesized different WO_3_ nanorod–graphene nanocomposites by varying the graphene weight ratio through a facile hydrothermal method. The electrochemical tests confirmed the superior energy storage performances of the composites with a graphene weight ratio of 1 wt%, which possesses a C_s_ of 343 F/g at a current density of 0.2 A/g, compared with a C_s_ of 300 F/g obtained for bare WO_3_ nanorods. The authors ascribed the superior performances of the nanocomposites to their high electron conductivity, but the concentration of active sites is also crucial. The energy storage dependence on the latter was studied by Cai et al. [64] who synthesized graphene nanosheet–WO_3_ nanocomposites by using a facile approach. The comparison between the energy storage performances of bare WO_3_ and of the graphene nanosheet–WO_3_ nanocomposites reveals an electrochemical superiority of the latter, which possesses a C_s_ of 140.8 F/g at 0.3 A/g, compared with 25.4 F/g obtained with bare WO_3_. The authors ascribed the high performances of the graphene nanosheet–WO_3_ nanocomposites to the interaction between WO_3_ and the nanosheets, especially at the edges of the latter, thus increasing the concentration of the available active sites. A similar interaction was also observed by Chu et al. [66], who compared the electrochemical performances of bare WO_3_ nanoflowers and of WO_3_ nanoflower–graphene nanosheet nanocomposites, thus obtaining 127 F/g and 495 F/g at 1 A/g, respectively. Additionally, the authors ascribed the best energy storage performances of the nanocomposites to the high concentration of electroactive sites and to the rapid electronic transport and short diffusion ion paths due to the optimal contact between WO_3_ and graphene nanosheets.

**Figure 7:**
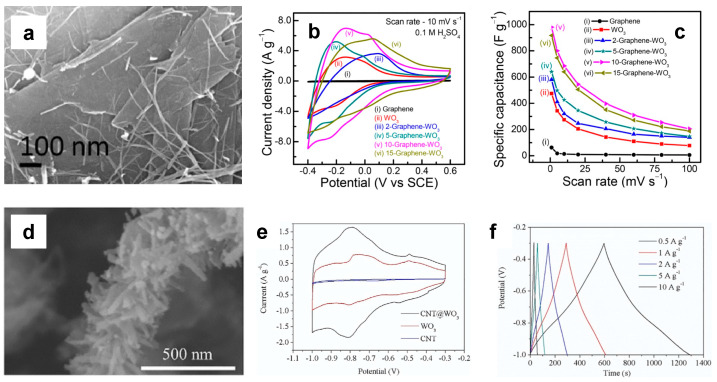
(**a**,**d**) SEM images, (**b**,**e**) CV and (**c**,**f**) Cs as a function of scan rate and GCD curves, of WO_3_–graphene nanocomposites [56], and WO_3_ nanorods on CNTs [67]. Reproduced with permission.

**Figure 8:**
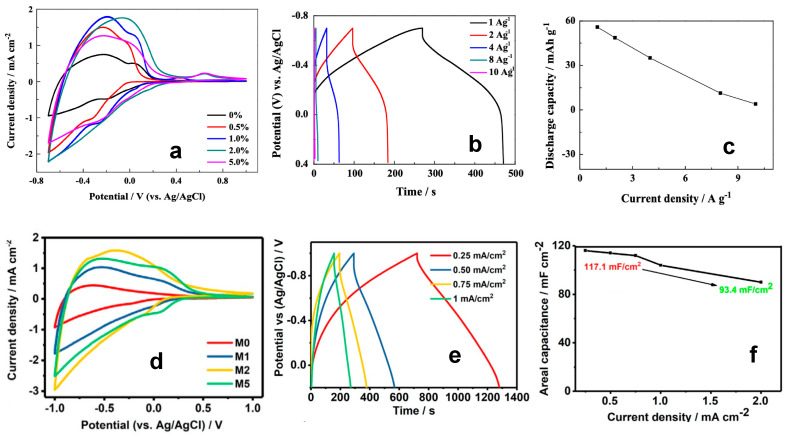
(**a**) CV curves of Mo-doped WO_3_ nanowires as a function of the Mo concentration, (**b**) GCD curves of electrodes obtained with 2% of Mo-doped WO_3_ nanowires, and (**c**) related discharge capacity as a function of current density [54]; (**d**) CV curves of Mo-doped WO_3_ thin film as a function of Mo concentration; (**e**) GCD curves of the electrodes obtained by using a composite in which the atomic ratio between Mo and W is equal to 0.02 (M2); (**f**) related areal capacitance as a function of current density [70]. Reproduced with permission.

Besides the development of WO_3_–graphene nanocomposites, many papers report the decoration of carbon nanotubes (CNTs) and multiwalled carbon nanotubes (MWCNTs) with WO_3_ nanorods or nanowires. Figure 7 shows the comparison between the electrochemical performances of WO_3_–graphene nanocomposites (Figure 7a) synthesized by Nayak et al. [56] and of WO_3_ nanorods on CNTs (Figure 8d), synthesized by Di et al. [67]. Both electrochemical tests were carried out in an H_2_SO_4_ electrolyte, and CV curves are characterized by a typical quasi-triangular shape (Figure 7b,e, respectively). Moreover, the CV curves of the WO_3_–graphene nanocomposites and the CNT@WO_3_ electrodes are characterized by the presence of WO_3_ redox peaks in the CV curves of the WO_3_–C nanocomposites which reveals the formation of stable junctions. Both Nayak’s and Di’s groups compared the electrochemical performances of the bare WO_3_ with those of the WO_3_–carbon nanocomposites, thus confirming the superiority of the latter. In particular, Nayak et al. [56] obtained a C_s_ of 1000 F/g at 10 mV/s compared to 500 F/g obtained for the bare WO_3_ nanowires. Di et al. [67] obtained a C_s_ of 496 F/g at 0.5 A/g for the CNT@WO_3_ composites. Table 2 reports a comparison between the most important energy storage parameters of many WO_3_–carbon-based nanocomposites.

State-of-the-art research related to the energy storage capabilities of WO_3_–carbon-based nanocomposites confirm the possibility of decisively improving the electronic conductivity of WO_3_. For this to happen, intimate contact between the WO_3_ and the carbon material is crucial, as well as an optimization of their relative composition. The presence of carbon-based materials increases the concentration of active sites, and their porosity allows for the reduction of the ion diffusion path, leading to an increase in the diffusion and surface limited current contribution during the energy storage mechanism. Despite the great results of this approach to increase electronic conductivity, another one was developed in recent years concerning the metal-doping of WO_3_ nanostructures, whose details will be reported in the next paragraph.

### 4.3. Metal-Doped WO_3_ Nanostructures for Supercapacitors

The low electron conductivity of WO_3_ limits its application in energy storage devices, despite the good electrochemical stability and affinity towards interaction with small ions, such as H^+^, Li^+^, and Na^+^. State-of-the-art research about the opportunities to improve the conduction of WO_3_ shows a great interest in the metal-doping of WO_3_ nanostructure procedure as a suitable method. The formation of interconnected structures in the bulk of doped WO_3_ reduces the ion diffusion path length by increasing the electron conductivity and the energy storage performance. The effect of several metals was studied as a function of the doping concentration. Kumar et al. [68] synthesized Co-doped nanoporous WO_3_ on CNTs. The energy storage activity of doped WO_3_ is analyzed as a function of the Co concentration in 2 M KOH electrolyte. CV analysis revealed that the electrode prepared by using 5% Co shows a weak increase of C_s_ (60 F/g at 1 A/g) compared with that of bare nanoporous WO_3_ (50 F/g at 1 A/g). Moreover, the authors demonstrated that an excess of Co may disturb the charge transport, thus leading to a lowering of the C_s_.

Among the analyzed metals, Sn is one of the most interesting, thanks to its effect on the electrochemical activity of WO_3_. Dharmalingam et al. [69] synthesized Sn-doped WO_3_ nanoplates and studied the role of the Sn concentration in morphology, crystal structure, and energy storage performances. The authors obtained remarkable energy storage performances in 1 M KOH for the electrode with the highest Sn concentration which possessed a C_s_ of 418 F/g at 1 A/g, compared with that of bare WO_3_ nanoplates (174 F/g at 1 A/g).

A great interest was aroused by Mo, thanks to its similar atomic structure to that of W. The Mo presence in the WO_3_ crystal induces lattice distortion which can facilitate the ion transport. State-of-the-art research reports the doping of both nanostructures and thin films, thus highlighting the role of Mo in energy storage behavior and activity as a function of concentration. Figure 8 shows the electrochemical comparison between Mo-doped WO_3_ nanowires, synthesized by Zhou et al. (tested in 0.5 M H_2_SO_4_) [54] and Mo-doped WO_3_ thin film, synthesized by Xie et al. (tested in 1 M LiClO_4_) [70]. In both cases, the storage activity is studied as a function of the Mo concentration, thus individuating the optimal concentration for the best energy storage activity. Despite the morphological differences, both Mo-doped WO_3_-based electrodes show similar quasi-triangular CV curves (Figure 8a,d), regardless of the Mo concentration and the electrolytes, thus revealing that Mo acts the same way, thus introducing the proper structure distortion. Additionally, the GCD curves show a similar trend (Figure 8b,e), thus further confirming the same energy storage mechanism which depends only on the Mo doping. Zhou et al. [54] obtained a discharge capacity of 55.89 mAh/g at 1 A/g for the sample with a 2% Mo concentration (Figure 8c), while Xie et al. [70] obtained an areal capacitance of 334.6 mF/cm^2^ at 0.25 mA/cm^2^ for the composite with the atomic ratio between Mo to W equal to 0.02 (Figure 8f).

State-of-the-art research on doping of WO_3_ confirms the possibility of increasing the electronic conductivity of WO_3_ nanostructures, thus lowering the length of the ion diffusion path. To this aim, other suitable candidates for WO_3_ doping are rare earths. Wang et al. [71] doped WO_3_ porous nanofilm with Ce, Eu, Sm, and Gd, and demonstrated that the Gd-doped WO_3_ possesses the highest areal capacitance (62.43 mF/cm^2^ at 0.3 mA/cm^2^), compared with that of bare WO_3_ (13.27 mF/cm^2^ at 0.3 mA/cm^2^). The authors ascribed the best performances of the rare-earth-doped WO_3_ to the facilitation of H^+^ ion insertion in the bulk of WO_3_ by the rare earth atoms.

A comparison between the energy storage performances of the most recent, state-of-the-art research about metal-doped WO_3_ nanostructures is reported in Table 3. Literature on metal-doped WO_3_ for energy storage applications confirms the possibility of improving electron conductivity with the help of the doping procedure, which leads to the introduction of lattice distortions. As demonstrated, storage performances strongly depend on the dopant nature and concentration, whereby careful and in-depth analysis is necessary each time.

## 5. WO_3_ Nanostructure-Based Devices

The state-of-the-art research about the application of WO_3_ nanostructures for energy storage demonstrates a great interest of the scientific community toward the exceptional properties of this material. Electrochemical activity strongly depends on the morphology and crystal structure, which can be tailored at the nanoscale thanks to the possibility of properly setting the synthesis parameters, as shown in the previous paragraphs. Moreover, many efforts have been made to further improve electronic conductivity, such as the development of WO_3_–carbon-based material nanocomposites or the metal-doping of WO_3_ nanostructures. The study of the electrode properties from the electrochemical point of view supports the realization of energy storage devices, such as symmetric and asymmetric supercapacitors (SSCs and ASCs, respectively), in which the WO_3_-based electrode acts as the anode. Then, in an SSC both the anode and the cathode are composed of the same electroactive material, while in an ASC the anode and cathode are different. In general, the coupling of suitable materials for the ASC configuration allows reaching higher performances than those of the SSC configuration. The determination of the potential range is crucial for the evaluation of the real energy storage performance of the device [72], whereby the anode and cathode are coupled depending on their operative potential range. In the case of an ASC, a wide potential range can be explored (1.5–2 V) to reach the maximum energy storage activity since both the cathode and anode are active in the different potential ranges [73]. For the realization of efficient energy storage devices, the electroactive mass of the anode and cathode must satisfy the charge balance principle, which ensures that the electrochemical charge stored at the cathode is equal to those produced at the anode and vice versa (Q_+_ = Q_−_) [40,41,42]:

(3) $$\frac{m_{-}\;}{m_{+}}=\frac{C_{s}{}_{+}\times \Delta V_{+}\;}{C_{s}{}_{-}\times \Delta V_{-}}$$

in which *m_−_*, *m_+_, C_S−_*, *C_S+_,* Δ*V_−_*, and Δ*V_+_* are the mass of the electrochemically active material, the specific capacitance obtained from CV analysis, and the potential interval in which the electrochemical tests are performed of the anode and the cathode, respectively. Moreover, energy and power densities are the most used parameters for the comparison of the performances of different energy storage devices and can be calculated from GCD curves as follow [51]:

(4) $$E_{d}=\frac{1}{2}C_{s}\Delta V^{2}$$

(5) $$P_{d}=E_{d}\Delta t$$

where $C_{s}$ is the specific capacitance obtained from the GCD curves, ΔV is the potential interval and Δt is the discharge time.

**Figure 9:**
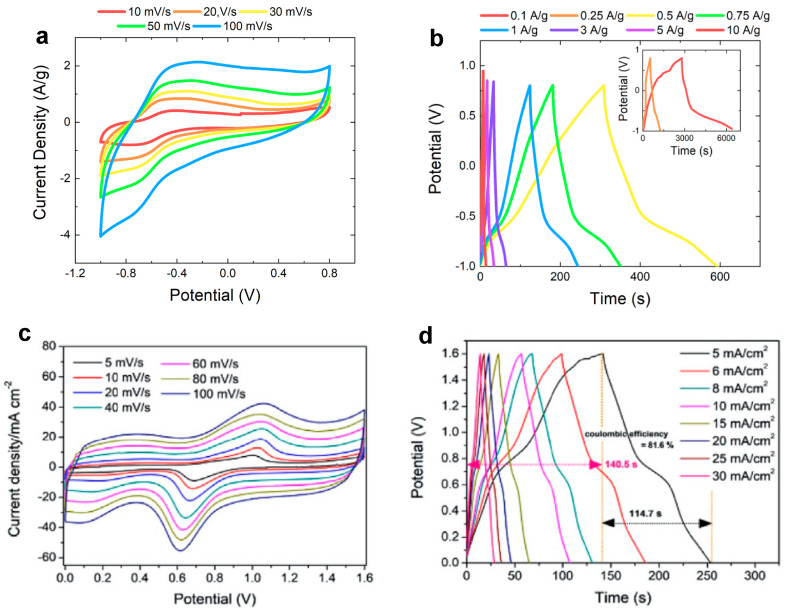
(**a**) CV curves of an ASC by using WO_3_ nanorods as anode and graphene paper as cathode and (**b**) related GCD curves [19]; (**c**) CV curves of an ASC by using WO_3_ nanorods as anode and RuO_2_ as cathode and (**d**) related GCD curves at different current densities [46]. Reproduced with permission.

Many examples of SSCs and ASCs implemented by using a WO_3_-based electrode as an anode are reported in the literature. Zheng et al. [51] studied the difference between an SSC and an ASC by creating WO_3_ nanofibers which act as the electroactive material in both devices and using active carbon as the positive electrode in the ASC. In both cases, the electrochemical analyses were conducted in 1 M Na_2_SO_4_ electrolyte in a potential region from 0 V to 1.8 V. The SSC devices show quasi-rectangular CV curves, while in the ASC an oxidation peak appears due to the role of the activated carbon. The authors obtained high energy densities of 99.0 and 88.2 Wh/kg at a power density of 450 W/kg for the SSC and ASC, respectively. Despite this result, state-of-the-art research about WO_3_-based energy storage devices reports a plethora of characterization of the ASC configurations, obtained using different materials, among which carbon-based composites are the most suitable. Nayak et al. [56] realized a solid-state ASC by using graphene–WO_3_ nanowire nanocomposites as the negative electrode and activated carbon as the positive electrode with H_2_SO_4_/PVA gel as solid electrolytes. They studied different potential windows and performed in-depth electrical analyses from 0 V to 2 V. The authors obtained an energy density of 26.7 Wh/kg at a power density of 6 kW/kg. Additionally, Shi et al. [43] report the application of activated carbon as the positive electrode in WO_3_-based ASC. The authors realized an asymmetric solid-state planar micro-supercapacitor by using hierarchical porous lignin-derived carbon (HPC)/WO_3_ nanostructures and active carbon as positive and negative electrodes, respectively, with PVA/H_2_SO_4_ gel electrolyte. The devices show an energy density of 34.2 Wh/kg at a power density of 237 W/kg and an energy density of 16 Wh/kg at a power density is 14,300 W/kg. Remarkable results are also obtained by coupling WO_3_ and RuO_2_ electrodes in an ASC configuration. Chang et al. [74] fabricated an ASC by using a WO_3_–WO_3_·0.5H_2_O nanorod mixture as the anode and RuO_2_·xH_2_O as the cathode. The operating voltage was 1.6 V, and the authors obtained energy and power densities of 23.4 Wh/kg and 5.2 kW/kg, respectively. Figure 9 reports the electrochemical comparison between ASCs realized by using WO_3_ nanorods as anodes in both cases and (a,b) graphene paper [19] and (c,d) RuO_2_ [46] as cathodes in 1M and 2 M H_2_SO_4_, respectively. CV curves of the WO_3_/RuO_2_ ASC (Figure 9c) show redox peaks that do not appear in the CV curves of the WO_3_/graphene paper ASC (Figure 9a), thus suggesting that they depend only on the RuO_2_ electrode. GCD curves (Figure 9b,d) show similar trends, regardless of the current densities, especially in the discharge region. WO_3_/graphene paper ASC shows the highest P_d_ of 9000 W/Kg at E_d_ of 18 Wh/kg [19], while the WO_3_/RuO_2_ ASC shows the highest power density of 540 W/kg at an energy density of 16.92 W h/kg, thus suggesting that the use of graphene paper as a cathode can improve the energy storage performances in terms of power and energy densities. Table 4 reports the electrochemical comparison between the state-of-the-art research on WO_3_-based ASCs and SSCs.

**Figure 10:**
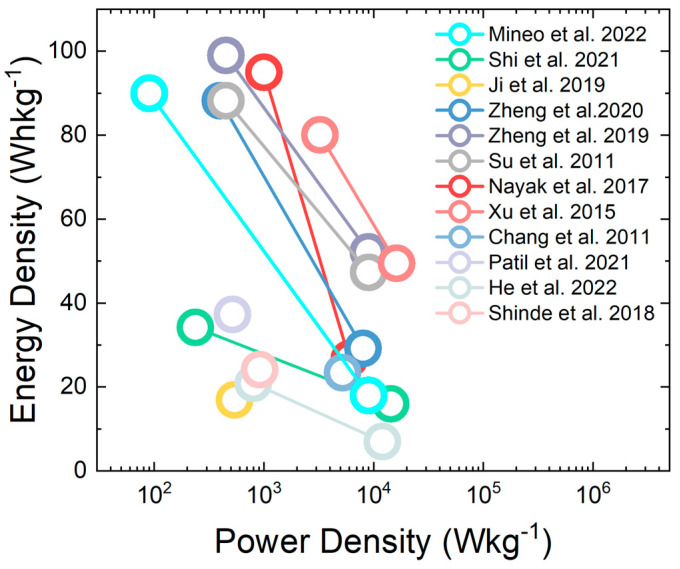
Ragone plot of the state-of-the-art research about WO_3_-based ASCs and SSCs. Reproduced by: Mineo et al., 2022 [19] Shi et al., 2021 [43], Ji et al., 2019 [46], Zheng et al., 2020 [50], Zheng et al., 2019 [51], Su et al., 2011 [53], Nayak et al., 2017 [56], Xu et al., 2015 [62], Chang et al., 2011 [74], Patil et al., 2021 [75], He et al., 2022 [76], Shinde et al., 2018 [77].

The Ragone plot is a meaningful representation of the energy storage activity of devices. Figure 10 reports the Ragone plot in which the WO_3_-based ASC and SSC are compared in terms of energy and power density, calculated from GCD curves at different current densities. The obtained values are in accordance with those obtained for pseudocapacitive electrodes used in ASC and SSC configurations. Most of the state-of-the-art data show high energy densities at remarkable power densities, thus confirming the excellent energy storage performances of WO_3_ nanostructures. Their high electrochemical stability and their pseudocapacitor behavior make them able to store high levels of quickly available charge, which allows for the supply of high power and energy density at the same time with a single device.

## 6. Conclusions and Outlooks

The finite supply of fossil fuels and environmental pollution have made the development of green energy sources and efficient energy storage systems in high demand. Electrochemical devices, such as batteries, electrochemical double layer capacitors, and pseudocapacitors, have aroused a great interest in the scientific community, thanks to the possibility of supplying different energy and power densities depending on the application. Among them, pseudocapacitors can store electric charges thanks to faradaic and adsorption reactions which occur at the surface of the electrochemically active material, thus supplying high energy and power density at the same time. Transition metal oxides are the most used materials for the realization of pseudocapacitors and, among them, WO_3_ is one of the most suitable choices, thanks to its high electrochemical stability, and earth abundance. In its crystalline form WO_3_ is composed of octahedra sharing corners and edges, and thanks to its high coordination number, it possesses many crystalline phases. Moreover, WO_3_ can be easily synthesized in the nanostructured form by using different low-cost synthesis techniques. In recent years WO_3_-based composites were analyzed in depth from the electrochemical point of view for energy storage applications, thus studying the role of morphology, crystal structure, and stoichiometry. Much progress has been made in this field, thus reaching interesting results, especially when hexagonal 1D WO_3_ nanostructures are used. The hexagonal structure facilitates the accommodation of small ions, such as H^+^, Li^+^, and Na^+^, in the hexagonal cavities. Despite the scientific community’s efforts, the electronic conductivity of WO_3_ remains low and affects energy storage performances. Different approaches were used to overcome this problem, such as the realization of WO_3_–carbon-based nanocomposites, in which, thanks to the carbon role, the energy storage performances are higher than those of the bare WO_3_ counterpart. A certain synergistic effect is revealed between WO_3_ and carbon-based material, which results in a higher electron conductivity. Another strategy used to solve the WO_3_ problems related to low conductivity is metal doping. The effects of this procedure depend on the nature and concentration of the doping metal, which introduces distortion in the WO_3_ lattice. This leads to a lowering of the ion diffusion path, resulting in increased performances of doped WO_3_ in comparison to the bare WO_3_.

Herein, a review of the recent results about WO_3_ nanostructure-based electrodes for energy storage applications is reported. After a brief excursion on the most important electrochemical analysis for the electrode characterization (Cycling Voltammetry, Galvanostatic Charge and Discharge, and Electrochemical Impedance Spectroscopy), the most common electrode preparation methods are reported from a critical point of view. The results on energy storage performances of WO_3_ nanostructures, of WO_3_/carbon-based nanocomposites, and of metal-doped WO_3_ nanostructures are summarized and described as a function of the role of morphology, crystal structure, and stoichiometry and metal-doping concentration. To understand the feasibility of WO_3_ electrodes for energy storage devices, a critical review of the most recent results about WO_3_-based symmetric and asymmetric supercapacitors is reported. State-of-the-art research results confirm the possibility of developing excellent WO_3_-based energy storage devices, with safe operation and developed by low-cost procedures, able to supply high energy and power densities.

The increasing energy demand requires low-cost, earth-abundant, safe, and efficient equipment, able to supply appropriate power and energy densities. WO_3_ demonstrated optimal energy storage performances despite its low electronic conductivity, which can be improved by careful tailoring the morphology, the crystal structure, and the stoichiometry. Different strategies were implemented to this aim, such as the metal-doping of WO_3_ and the development of hybrid WO_3_–carbon-based nanocomposites. The latter seems to be the most promising approach since these hybrid structures, in addition to being able to be made with low-cost methods, show very high specific capacitance values and remarkable power and energy densities for next-generation devices.

## Figures and Tables

**Table 1:** State-of-the-art on energy storage applications of WO_3_ nanostructure-based electrodes.

Morphology	Electrolyte	Potential Interval	C_s_	Ref.
Nanorods and urchin-like nanostructures	1 M H_2_SO_4_	−0.5 V ÷ 0 V	632 F/g @ 5 mV/s 466 F/g @ 0.5 A/g	[19]
Nanocubes	1 M H_2_SO_4_	−0.6 V ÷ 0.2 V	377 F/g @ 2 mV/s	[42]
Nanoplates	0.5 M H_2_SO_4_	−0.3 V ÷ 0.2 V	334 F/g @ 2 mV/s	[44]
Nanotubes on nanoplates	0.5 M H_2_SO_4_	−0.3 V ÷ 0.2 V	2552 mF/cm^2^ @ 1 mA/cm^2^	[45]
Nanorods	2 M H_2_SO_4_	−0.6 V ÷ 0.2 V	900 F/g @ 3 mV/s	[46]
Nanoflakes	1 M NaSO_4_	−0.1 V ÷ 0.8 V	588 F/g @ 5 mV/s 538 F/g @ 0.1 A/g	[50]
Nanorods	1 M H_2_SO_4_	−0.65 V ÷ 0.2 V	538 F/g @ 5 mV/s 425 F/g @ 2 mA/cm^2^	[57]
Nanowires	1 M H_2_SO_4_	−0.4 V ÷ 0.4 V	500 F/g @ 10 mV/s	[60]
Nanotubes	0.5 M H_2_SO_4_	−0.7 V ÷ 0 V	600 F/g @ 3 mV/s	[62]
Microspheres	2 M H_2_SO_4_	−0.35 V ÷ 0.2 V	872 F/g @ 10 mV/s 797 F/g @ 0.5 A/g	[63]

**Table 2:** Recent, state-of-the-art progress on energy storage applications of WO_3_–carbon nanocomposite-based electrodes.

Morphology	Electrolyte	Potential Interval	Cs	Ref.
HPCO/WO_3_	1 M H_2_SO_4_	−1 V ÷ 0.4 V	432 F/g @ 0.5 A/g	[43]
Graphene-supported WO_3_ nanowires	0.1 M H_2_SO_4_	−0.4 V ÷ 0.6 V	1000 F/g @ 10 mV/s	[56]
Graphene sheet/WO_3_	1 M H_2_SO_4_	0 V ÷ 1 V	148 F/g @ 0.3 A/g	[64]
Graphene-supported WO_3_ nanorods	0.5 M H_2_SO_4_	−0.5 V ÷ 0 V	343 F/g @ 0.2 A/g	[65]
Graphene sheet/WO_3_ nanoflowers	0.5 M H_2_SO_4_	−0.4 V ÷ 0.3 V	495 F/g @ 1 A/g	[66]
CNTs/WO_3_ nanorods	0.5 M H_2_SO_4_	−1 V ÷ 0.3 V	496 F/g @ 0.5 A/g	[67]

**Table 3:** State-of-the-art on energy storage applications of metal doped WO_3_-based electrodes.

Morphology	Electrolyte	Potential Interval	Cs	Ref.
Mo-doped WO_3_ nanowires	0.5 M H_2_SO_4_	−0.7 V ÷ 1 V vs. Ag/AgCl	55.88mAh/g @ 1 A/g	[54]
Co-doped WO_3_@CNTs	2 M H_2_SO_4_	0 V ÷ 0.6 V vs. Hg/HgO	60 F/g @ 1 A/g	[68]
Sn-doped WO_3_ nanoplates	1 M KOH	0 V ÷ 0.55 V vs. Ag/AgCl	418 F/g @ 1 A/g	[69]
Mo-doped WO_3_ thin films	1 M LiClO_4_	−1 V ÷ 1 V vs. Ag/AgCl	334.6 mF/g @ 0.25 mA/cm^2^	[70]
Gd-doped WO_3_ nanoflowers	0.3 M HCl	−0.6 V ÷ 0.6 V vs. Ag/AgCl	79.52 mF/cm^2^ @ 0.3 mA/cm^2^	[71]

**Table 4:** State-of-the-art on energy storage applications of WO_3_-based ASCs and SSCs.

Configuration	Electrodes	Electrolyte	Potential Interval	Cs	Ref.
ASC	WO_3_ nanorods graphene paper	1 M H_2_SO_4_	−1 V ÷ 0.8 V	90 Wh/kg @ 90 W/Kg 18 Wh/kg @ 9000 W/Kg	[19]
ASC	HPCO/WO_3_ activated carbon	H_2_SO_4_/PVA Solid gel	0 V ÷ 1 V	34.2Wh/kg @ 237 W/Kg 16 Wh/kg @ 14,300 W/Kg	[43]
ASC	WO_3_ nanorods RuO_2_	2 M H_2_SO_4_	0 V ÷ 1.8 V	16.9 Wh/kg @ 540 W/Kg	[46]
SSC	WO_3_ nanofibers	1 M Na_2_SO_4_	0 V ÷ 1.8 V	99 Wh/kg @ 450 W/Kg	[51]
ASC	WO_3_ nanofibers activated carbon	1 M Na_2_SO_4_	0 V ÷ 1.8 V	88.2 Wh/kg @ 450 W/Kg	[51]
ASC	Graphene–WO_3_ nanowires	H_2_SO_4_/PVA Solid gel	0 V ÷ 2 V	26.7 Wh/kg @ 6000 W/Kg	[56]
ASC	WO_3_-WO_3_·0.5H_2_O nanorods RuO_2_·H_2_O	0.5 M H_2_SO_4_	−1.6 V ÷ 0 V	24 Wh/kg @ 5200 W/Kg	[74]

## Data Availability

The data presented in this study are available upon request from the corresponding author.
